# Silibinin Inhibits Platelet-Derived Growth Factor-Driven Cell Proliferation via Downregulation of N-Glycosylation in Human Tenon's Fibroblasts in a Proteasome-Dependent Manner

**DOI:** 10.1371/journal.pone.0168765

**Published:** 2016-12-28

**Authors:** Yi-Hao Chen, Ching-Long Chen, Da-Wen Lu, Chang-Min Liang, Ming-Cheng Tai, Jiann-Torng Chen

**Affiliations:** 1 Graduate Institute of Medical Science, National Defense Medical Center, Taipei, Taiwan; 2 Department of Ophthalmology, Tri-Service General Hospital, National Defense Medical Center, Taipei, Taiwan; Centre de Recherche en Cancerologie de Lyon, FRANCE

## Abstract

The objective of this study was to evaluate the effects of silibinin on cell proliferation in platelet-derived growth factor (PDGF)-treated human Tenon's fibroblasts (HTFs). The effect of silibinin on cell proliferation in PDGF-treated HTFs was determined by examining the expression of proliferating cell nuclear antigen (PCNA) and performing WST-1 assays. Cell cycle progression was evaluated using flow cytometry. The related cyclins and cyclin-dependent kinases (CDKs) were also analyzed using western blot. A modified rat trabeculectomy model was established to evaluate the effect of silibinin on cell proliferation in vivo. Western blot analysis was carried out to determine the effect of silibinin on the expression of PDGF receptor and on the downstream signaling pathways regulated by PDGF receptor. PDGF elevated the expression of PCNA in HTFs, and this elevation was inhibited by silibinin. The inhibitory effect of silibinin on cell proliferation was also confirmed via WST-1 assay. PDGF-stimulated cell cycle in HTFs was delayed by silibinin, and the related cyclin D1 and CDK4 were also suppressed by silibinin. In the rat model of trabeculectomy, silibinin reduced the expression of PCNA at the site of blebs in vivo. The effects of silibinin on PDGF-stimulated HTFs were mediated via the downregulation of PDGF receptor-regulated signaling pathways, such as ERKs and STATs, which may be partially caused by the downregulation of N-glycosylation of PDGF receptor beta (PDGFRβ). The effect of silibinin on modulation of N-glycosylation of PDGFRβ was mediated in a proteasome-dependent manner. Silibinin inhibited cell proliferation and delayed cell cycle progression in PDGF-treated HTFs in vitro. PDGF also modulated the process of N-glycosylation of the PDGFRβ in a proteasome-dependent manner. Our findings suggest that silibinin has potential therapeutic applications in glaucoma filtering surgery.

## Introduction

Glaucoma is a major cause of irreversible blindness worldwide and presents as a progressive optic atrophy [1–3]. Until date, lowering intraocular pressure (IOP) has been the only successful therapeutic strategy for treating glaucoma. The most common surgical treatment for glaucoma is the procedure known as glaucoma filtering surgery [4]. Glaucoma filtering surgery is performed to create an artificial route to drain the aqueous humor from the anterior chamber to the subconjunctival space, leaving a bleb that is formed in the subconjunctival space [5]. The bleb formation is similar to the wound healing process of soft tissues, which involves inflammation, proliferation, and wound remodeling [6, 7]. Scar formation in the bleb, which is a manifestation of extensive wound healing process, is the major cause of failure in this surgery [8]. To attenuate scar formation, the inhibition of inflammation, proliferation, or wound remodeling has been proposed as a strategy. Several agents have been studied for adjunctive use [9, 10], but none has produced satisfactory results.

Several growth factors are involved in the wound healing process, and one of them is the platelet-derived growth factor (PDGF), a major mitogen in the early developmental stages [11, 12]. The PDGF signaling network consists of four ligands, PDGF A-D, and two receptors, PDGF receptor alpha (PDGFRα) and PDGFRβ, which belong to the receptor tyrosine kinases (RTKs) family of receptors [13]. When PDGF is secreted, disulfide-linked homo- or hetero-dimers are formed. PDGF stimulates chemotaxis of neutrophils and macrophages during inflammation [14]. PDGF and its receptor have been shown to be upregulated in the wound area and are required for fibroblast proliferation and differentiation [15]. PDGF has also been shown to participate in the wound healing process of trabeculectomy blebs [11]. Therefore, inhibiting the activation of PDGF-PDGFR signaling was hypothesized as a possible strategy to decrease the scar formation in the bleb.

Silibinin, the major component of the silymarin complex, is extracted from milk thistle. Traditionally, it is widely used in the treatment of liver diseases such as hepatitis, liver cirrhosis, and alcoholic liver diseases [16]. Furthermore, it exerts strong anticancer effects in various cancer cells including that of the prostate, skin, breast, colon, lung, and kidney. The inhibitory effects of silibinin against cancer cells include inhibition of proliferation, anti-inflammation, cell cycle regulation, apoptosis induction, inhibition of angiogenesis, and inhibition of epithelial–mesenchymal transition [17–19]. Our previous studies have shown that silibinin has an anti-inflammatory effect in ocular inflammatory disorder and an inhibitory effect on transforming growth factor-β regulated responses [20, 21]. Therefore, to develop novel approaches for decreasing bleb scarring, we investigated the inhibitory effects of silibinin on the proliferation of human Tenon's fibroblasts (HTFs), an expansion culture of the human Tenon's explants.

Further, we evaluated the effect of silibinin on PDGF-stimulated HTFs. Our studies revealed that silibinin inhibited the PDGF-stimulated cell proliferation and delayed the cell cycle. The inhibitory effects of silibinin were mediated through blockage of the PDGFR-regulated extracellular signal-regulated kinase (ERK) and signal transducer and activator of transcription (STAT) signaling pathways. In addition, silibinin exerted its effects via modulation of N-linked glycosylation of the PDGFRβ in a proteasome-dependent manner. Our study provided insights into the effects of silibinin on PDGF regulated cell proliferation and elucidated a possible mechanism of hypo-glycosylation of the PDGFRβ by silibinin.

## Materials and Methods

### Cells

Small Tenon's samples were biopsied during the standard intraocular procedures. Primary HTFs were obtained from the samples as described previously [22]. The regulations of the Declaration of Helsinki were followed, and the procedures were approved by the institutional ethics committee of the Tri-Service General Hospital, Taiwan. Informed consents were obtained from all participants. The cells were confirmed via immunofluorescence staining of α-smooth muscle actin. HTFs were propagated in Dulbecco’s modified Eagle’s medium (DMEM-F12; Gibco, Carlsbad, CA, USA) supplemented with 10% fetal bovine serum (FBS, Gibco, Carlsbad, CA, USA), 100 U/mL penicillin, and 100 μg/mL streptomycin (Sigma-Aldrich, St. Louis, MO, USA). Cells were maintained in the logarithmic growth phase. For all experiments, cells from 3–6 passages were used.

### Reagent

Silibinin (catalog no. S0417) was purchased from Sigma-Aldrich (St. Louis, MO, USA). The reagent was dissolved in dimethyl sulfoxide (DMSO) at 0.1M.

### Detection of cellular toxicity

Cytotoxicity of silibinin-treated cells was determined using a double-staining method with the fluorescein isothiocyanate (FITC)-labeled annexin-V (Invitrogen, Carlsbad, CA, USA) and propidium iodide (PI; Sigma-Aldrich, St. Louis, MO, USA). After treatment with various concentrations of silibinin for 24 h, the cells were trypsinized and counted, a fraction of the cells (2 ×10^5^) was collected by centrifugation, and the pellet was washed twice with PBS. After further centrifugation, the cell pellet was resuspended and incubated for 15 min in 100 μL of labeling solution [5 μL of annexin-V in 100 μL of HEPES buffer (10 mM HEPES/NaOH, pH 7.4, 140 mM NaCl, 5 mM CaCl_2_)]. Then, the cell suspension was collected by centrifugation and washed twice with PBS. Finally, 400 μL of HEPES buffer containing 2.5 μL of PI (50 μg/mL) was added and samples were analyzed on a FACScan flow cytometer (BD Biosciences, Franklin Lakes, NJ, USA).

### WST-1 cell proliferation assays

The cell proliferation assay was based on the ready-to-use cell proliferation reagent WST-1 (Clontech, Mountain View, CA, USA). After 24-h treatment with various concentrations of silibinin, 20 ng/mL PDGF was added to the medium in each well. After 48 h, 10 mL of WST-1 reagent was added to the medium in each well. The cells were incubated in a humidified atmosphere at 37°C in 5% CO_2_/95% air for 1 h. The multi-titer plates were then thoroughly shaken for 1 min and absorbance was determined at 450 nm. The background absorbance was measured on wells containing only the dye solution and the culture medium. Cell proliferation data were obtained from at least three experiments with at least six wells for each concentration in separate 96-well plates. The mean optical density values obtained for the untreated controls were assigned a value of 100%. The results are expressed as a percentage of the optical density of treated vs. untreated controls.

### Preparation of whole cell lysates

Confluent cultured cells were preincubated with or without tunicamycin (T7765, Sigma-Aldrich, St. Louis, MO, USA), chloroquine diphosphate salt (CQ; Sigma-Aldrich, St. Louis, MO, USA), benzyloxycarbonyl-Val-Ala-Asp-fluoromethyl ketone (Z-VAD; EMD Millipore, Billerica, MA, USA), or MG132 (Sigma-Aldrich, St. Louis, MO, USA) for 18 h. The cells were then incubated with or without 50 or 100 μM silibinin for 24 h before stimulation with 20 ng/mL PDGF (PeproTech, Rocky Hill, NJ, USA) for 24 h. Treated and untreated cells were washed with PBS, harvested by scraping, and centrifuged at 1000 *g*. Cell pellets were resuspended and sonicated in cold lysis buffer (PRO-PREPTM Protein Extraction Solution; iNtRON Biotechnology, Korea). The lysates were centrifuged at 12,000 *g* for 10 min, and the protein concentration in the clear supernatant was determined using BCA protein assay kit (Thermo Scientific Pierce, Rockford, IL, USA).

### Western blot analysis

Equal amounts of proteins (20 μg) were resolved using 10% SDS-PAGE and transferred to PVDF membranes. The membranes were blocked with 5% (w/v) milk for 1 h at room temperature and subsequently incubated for 1 h at room temperature with a 1:1000 dilution of antibodies against GAPDH (Rockland, Limerick, PA, USA), α-tubulin (Santa Cruz Biotechnology, Santa Cruz, CA, USA), actin (Gene Tex, Irvine, CA, USA), proliferating cell nuclear antigen (PCNA; BD Transduction Laboratories, San Diego, CA, USA), lamin B1 (Abcam, Cambridge, UK), phosphorylated STAT1 (Santa Cruz Biotechnology, Santa Cruz, CA, USA), total STAT1 (Santa Cruz Biotechnology, Santa Cruz, CA, USA), phosphorylated STAT3 (Santa Cruz Biotechnology, Santa Cruz, CA, USA), total STAT3 (Santa Cruz Biotechnology, Santa Cruz, CA, USA), phosphorylated ERK (Santa Cruz Biotechnology, Santa Cruz, CA, USA), total ERK (Santa Cruz Biotechnology, Santa Cruz, CA, USA), cyclin D1 (BD Transduction Laboratories, San Diego, CA, USA), cyclin-dependent kinase 4 (CDK4; BD Transduction Laboratories, San Diego, CA, USA), or PDGFRβ (Santa Cruz Biotechnology, Santa Cruz, CA, USA). The membranes were washed and incubated with horseradish peroxidase-conjugated secondary antibody (1:1000; Jackson ImmunoResearch Laboratories, West Grove, PA, USA) for 1 h at room temperature, and the proteins were visualized using an enhanced chemiluminescence procedure (enhanced chemiluminescence reagent, Millipore, Billerica, MA, USA).

### Cell cycle analysis

HTFs were treated with or without silibinin for 24 h in a serum-free medium and then released from arrest by incubation in a drug-free culture medium containing 20 ng/mL PDGF. Cells were then harvested with trypsin at various time points within 48 h, washed twice with PBS, fixed in ice-cold 70% (v/v) ethanol, and stored at 4°C until use. Before flow cytometric analysis, cells were washed with PBS, centrifuged, and the cell pellets were resuspended in RNase (1 mg/mL) for 30 min. Cells were then stained for 15 min with PI in PBS (final concentration 40 μg/mL) before analysis with a FACScan flow cytometer using CellQuest software (BD Biosciences, Franklin Lakes, NJ, USA).

### Animal model and immunohistological analysis

We conducted a modified trabeculectomy procedure on the eyes of SD rats weighing 160–200 g. Surgery was performed only on the right eye. The rats were anesthetized with an intraperitoneal injection of a combination of 50 mg/kg ketamine and 10 mg/kg xylazine. A topical anesthetic, 0.5% proparacaine eye drop (ALCAINE, Alcon, Vilvorde, Belgium), was also administered. After an eyelid speculum retracted the eyelids, a fornix-based conjunctival flap was created in the superior lateral quadrant of the eye at the limbus. Blunt dissection helped undermine the conjunctiva and the Tenon’s capsule. Next, a scleral tunnel was created using a 27-gauge needle. At this point, the aqueous humor could be observed to leak from the tunnel. Next, the conjunctival wound was closed with a running suture of 10–0 nylon at the limbus in a watertight manner. Finally, a sub-conjunctival injection with or without silibinin (100 μM) was administered at the surgical site to the experimental and control groups, respectively. Epi-scleral tissues at the site of the trabeculectomies were trimmed and ground for western blot analysis and the whole eyes were prepared for immunohistological examination 7 days after surgery. Eyeballs were enucleated, fixed in 10% neutral buffered formalin solution for 24 h, dissected at the equator, and embedded in paraffin. Sagittal sections of the anterior segments of the eyes containing the cornea, sclera, and conjunctiva were cut at a thickness of 5 μm, mounted on galectin-coated slides, and dried. Tissue sections were immersed in 3% hydrogen peroxide for 10 min to suppress endogenous peroxidase activity. Antigen retrieval was performed by heating each section at 100°C for 30 min in 10 mM citrate buffer containing 0.05% Tween 20 (pH 6.0). After three rinses of 5 min each in phosphate-buffered saline (PBS), the samples were blocked with 3% bovine serum albumin in phosphate-buffered saline for 1 h and then incubated with anti-PCNA monoclonal antibody as the primary antibody overnight at 4°C. After three washes of 5 min each in phosphate-buffered saline (PBS), the sections were incubated with Super Enhancer (BioGenex, San Ramon, CA, USA) for 20 min and then incubated with poly-horseradish peroxidase (BioGenex, San Ramon, CA, USA) as the secondary antibody for 30 min. After three additional washes, peroxidase activity was developed with diaminobenzidine (DAB) at room temperature. All experimental procedures were approved by the Animal Care and Use Committee of the National Defense Medical Center and were conducted in accordance with the Statement for the Use of Animals in Ophthalmic and Vision Research of the Association for Research in Vision and Ophthalmology.

### Fluorescence-based PDGF binding assay

HTFs were treated with or without silibinin or tunicamycin for 24 h in a serum-free medium and harvested with trypsin. Next, the cells were centrifuged at 500 *g* for 5 min, washed twice with PBS, and resuspended in PBS to a final concentration of 4 × 10^6^ cells/ml. Ten microliters of biotinylated rhPDGF-BB reagent (R&D System, Minneapolis, MN, USA) was added to 25 μL of the washed cell suspension in a 12 × 75 mm tube for a total reaction volume of 35 μL. After 30–60 min incubation of cells at 2–8°C, 10 μL of avidin-FITC reagent was added to each tube. The reaction mixture was then incubated for an additional 30 min at 2–8°C in the dark. Finally, the cells were washed twice with 2 mL of 1× cell wash buffer and resuspended in 0.2 mL of 1× cell wash buffer for flow cytometric analysis (BD Biosciences, Franklin Lakes, NJ, USA).

### Statistical methods

Normally distributed continuous variables were compared via one-way analysis of variance. When a significant difference between the groups was apparent, multiple comparisons of the means were performed using the Student-Newman-Keuls procedure. The data are presented as the means ± SE. Each result is representative of at least three independent experiments. All statistical assessments were two-sided and evaluated at the 0.05 level of significance.

## Results

### Effect of silibinin on HTF toxicity

**Fig 1:**
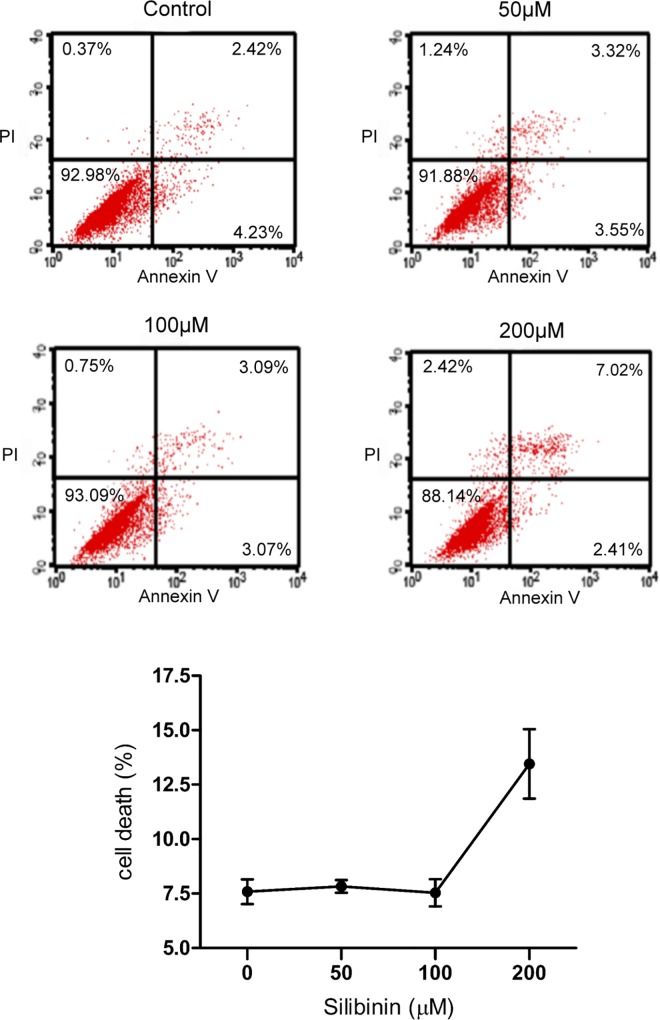
Cytotoxicity in human Tenon's fibroblasts (HTFs) treated with silibinin determined using flow cytometric analysis. The percentages of dead HTFs treated with 0, 50, 100 μM, and 200 μM silibinin were determined.

To assess cellular toxicity after silibinin treatment in HTFs, cell death was quantified using flow cytometry assay. Cells positive for either annexin-V or PI stains were considered dead cells. As shown in Fig 1, the population of cells increased after 24 h treatment with 200 μM silibinin. This finding revealed that the concentration of silibinin < 100 μM was non-toxic in HTFs. For further evaluation in our studies, 50 and 100 μM of silibinin were selected.

### Effect of silibinin on PDGF induced HTF proliferation

**Fig 2:**
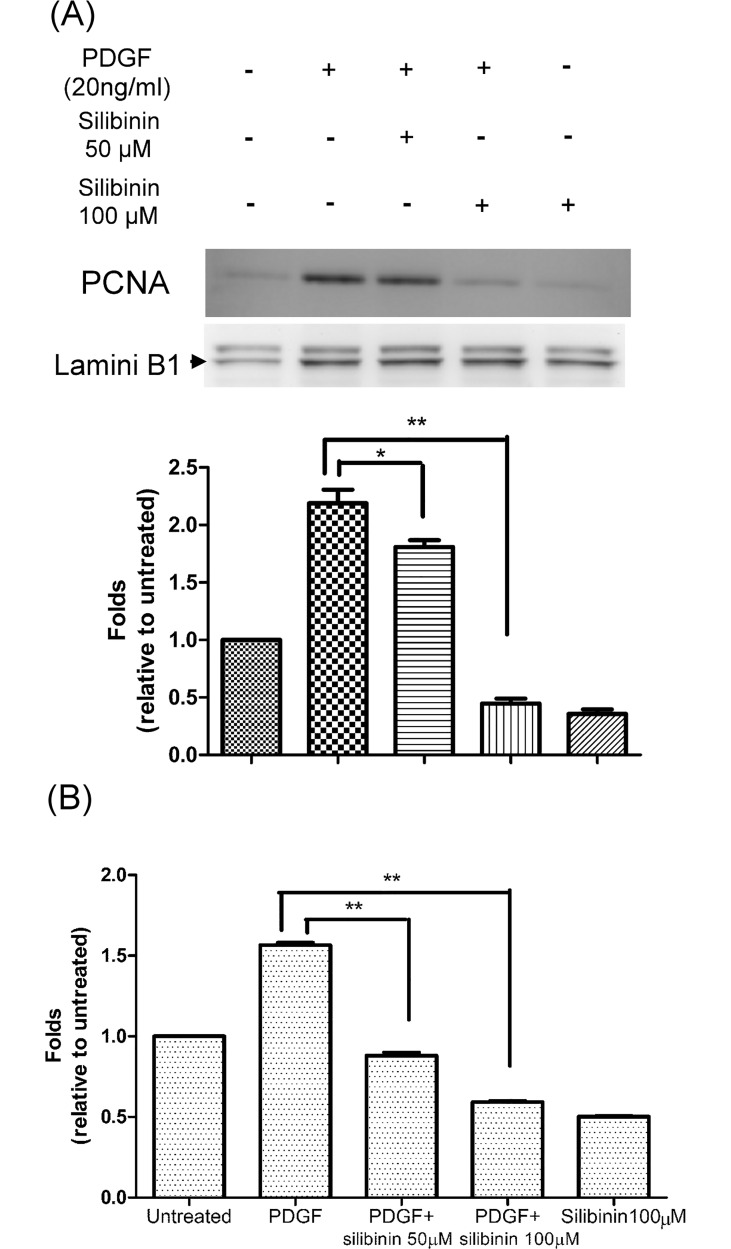
Effects of silibinin on cell proliferation in platelet-derived growth factor (PDGF) treated human Tenon's fibroblasts (HTFs). (A) HTFs were pretreated with either vehicle or silibinin (50 and 100 μM) for 24 h. The cells were then treated for 24 h with PDGF. Whole cell lysates were prepared and analyzed via western blotting by using antibodies directed against proliferating cell nuclear antigen (PCNA) and Lamin B1. Mean PCNA levels were measured using densitometric analysis and normalized to Lamin B1. (B) HTFs were pretreated with either vehicle or silibinin (50 or 100 μM) for 24 h. The cells were then treated with PDGF for 48 h. WST-1 assays were used to assess cell proliferation. Absorbance was measured and normalized to control (no silibinin). The data are presented as means ± SEM of three independent experiments. Asterisks (* and **) indicate responses that are significantly different (p < 0.05 and p < 0.01, respectively).

Western blot analysis with antibodies for PCNA, which is a DNA clamp that is essential for replication and also a marker of cell proliferation [23], and WST-1 proliferation assays were used to determine the effect of silibinin on proliferation of HTFs. As shown in Fig 2A, HTFs co-cultured with PDGF showed a significantly increased PCNA expression, which could be reversed by silibinin at 50 and 100 μM. Similar findings were observed in the WST-1 assays. Compared with the untreated group and the silibinin only group, PDGF stimulated the absorbance of the dye in HTFs, and silibinin decreased this effect of PDGF in a dose-dependent manner (Fig 2B). Findings from both the western blot and the WST-1 assay provided evidence for the inhibitory effect of silibinin on PDGF-stimulated proliferation of HTFs.

### Effect of silibinin on PDGF induced cell cycle progression

**Fig 3:**
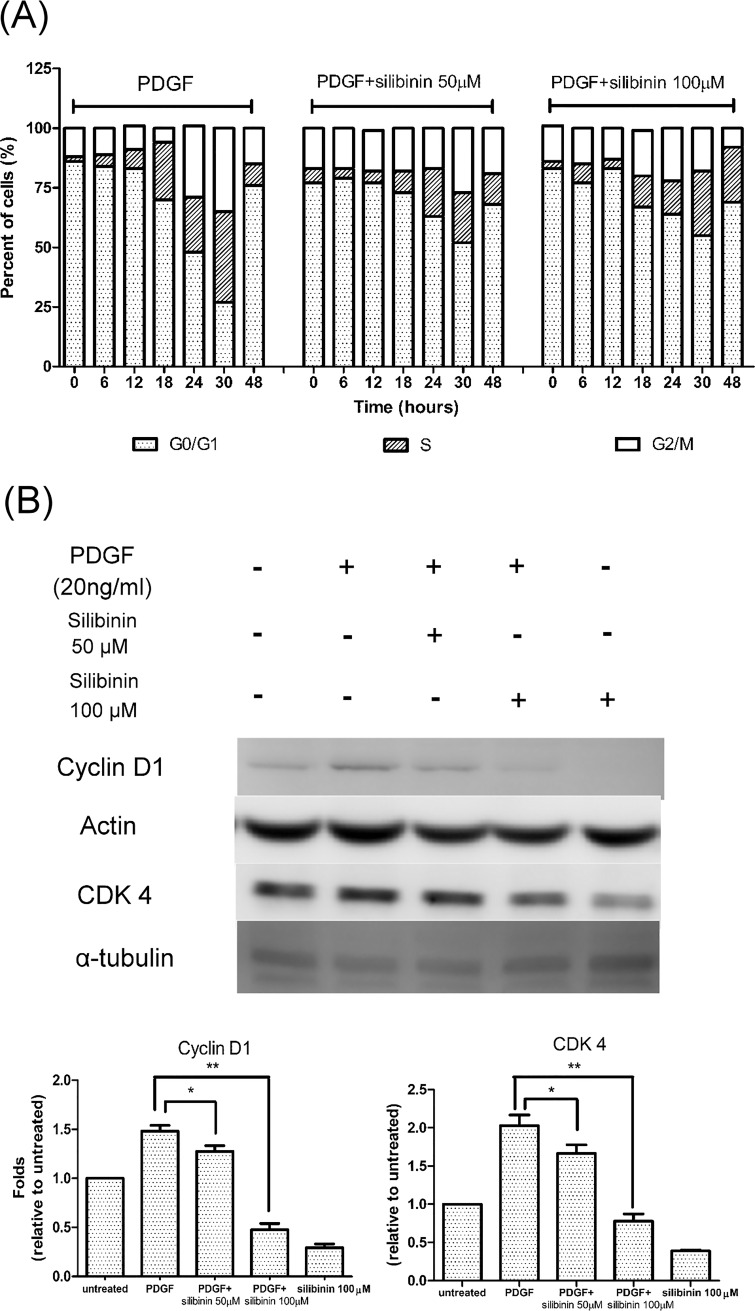
Effects of silibinin on platelet-derived growth factor (PDGF)-induced cell cycle progression in human Tenon's fibroblasts (HTFs). (A) HTFs were serum starved for 24 h and then harvested at the indicated times after stimulation with PDGF with or without the addition of silibinin. The percentage of cells in the G0-G1, S, and G2-M phases was determined using flow cytometry. (B) HTFs were pretreated with either vehicle or silibinin (50 or 100 μM) for 24 h. The cells were then treated with PDGF for 30 min. Whole cell lysates were prepared and analyzed via western blot by using antibodies directed against cyclin D1, actin, cyclin-dependent kinase 4 (CDK4), and α-tubulin. Mean cyclin D1 levels were determined using densitometric analysis and normalized to actin. Mean CDK4 levels were also determined using densitometric analysis and normalized to α-tubulin. The data are presented as means ± SEM of three independent experiments. Asterisks (* and **) indicate responses that are significantly different (p < 0.05 and p < 0.01, respectively).

We next examined the effects of silibinin on the progression of HTFs through the cell cycle after the cells had been synchronized in the G0-G1 phase. At 18 h after stimulation with PDGF, ~24% of untreated HTFs had entered the S phase compared with ~11% of the cells treated with 50 μM silibinin and ~13% of the cells treated with 100 μM silibinin. At 24 h and 30 h, ~30% and ~35% of the control cells progressed to the G2-M phase, respectively, compared with ~18% and ~27% of the cells treated with 50 μM silibinin and ~ 20% and ~18% of the cells treated with 100 μM silibinin. Even at 48 h, ~23% of the cells treated with 100 μM silibinin remained in the S phase, and very few (<10%) had entered the G2-M phase (Fig 3A). Cyclins and CDKs determine a cell's progression through the cell cycle. To further evaluate the effect of silibinin on the expression of cyclin and CDK, western blot analysis with antibodies for cyclin D1 and CDK4 was conducted. As shown in Fig 3B, the expression of cyclin D1 and CDK4 increased in HTFs after PDGF stimulation for 18 h. However, the amount of cyclin D1 and CDK4 significantly decreased in HTFs treated with both silibinin and PDGF. These findings showed that silibinin delayed PDGF-induced cell cycle progression in HTFs through modulation of cyclin D1 and CDK4.

### Anti-proliferative effect of silibinin in animal model

**Fig 4:**
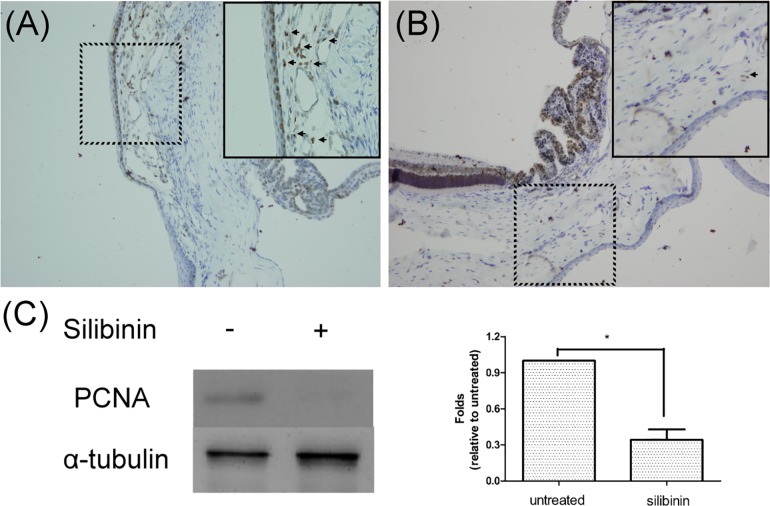
Effect of silibinin in the modified rat model of trabeculectomy. The bleb tissue from rats in the control group (no silibinin) (A) and the treatment group (injected with silibinin) (B) of the experimental rat trabeculectomy model was prepared. Immunohistochemistry images of proliferating cell nuclear antigen (PCNA) expression (arrow) in the bleb tissue are presented (the magnified images of the area defined by the dotted lines are shown in the corner of each picture). A suppressive effect of silibinin on PCNA expression was noted, which was also confirmed via western blot analysis (C).

To evaluate whether the anti-proliferative effect of silibinin observed in the cultured cells could be validated in an animal model, we injected silibinin into the subconjunctival space near the surgical wound in the rat model of trabeculectomy. Immunohistochemical staining was used to identify the differences in PCNA expression in the subconjunctival tissues. On day 7, the expression of PCNA in the site treated with silibinin (Fig 4B) decreased compared with that in sites without silibinin (Fig 4A). Moreover, the expression of PCNA was significantly downregulated by a local silibinin injection in the bleb tissues prepared for quantitative analysis by western blot (Fig 4C).

### Effect of silibinin on PDGF regulated signaling pathways

**Fig 5:**
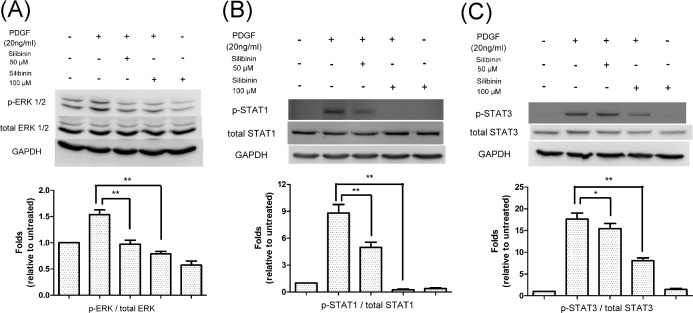
Effects of silibinin on platelet-derived growth factor (PDGF)-regulated signaling pathways in Human Tenon's fibroblasts (HTFs). HTFs were pretreated with either vehicle or silibinin (50 or 100 μM) for 24 h. The cells were then treated with PDGF for 30 min. (A) Whole cell lysates were prepared and analyzed via western blot by using antibodies directed against phosphorylated-extracellular-signal-regulated kinase (ERK) 1/2, total ERK 1/2, and GAPDH. Mean phosphorylated-ERK 1/2 levels were determined using densitometric analysis and normalized to total ERK 1/2. (B) Whole cell lysates were prepared and analyzed via western blot by using antibodies directed against phosphorylated-signal transducer and activator of transcription 1 (STAT1), total STAT1, and GAPDH. Mean phosphorylated-STAT1 levels were determined using densitometric analysis and normalized to total STAT1. (C) Whole cell lysates were prepared and analyzed via western blot by using antibodies directed against phosphorylated-STAT3, total STAT3, and GAPDH. Mean phosphorylated-STAT3 levels were determined using densitometric analysis and normalized to total STAT3. The data are presented as means ± SEM of three independent experiments. Asterisks (* and **) indicate responses that are significantly different (p < 0.05 and p < 0.01, respectively).

We assessed whether silibinin interferes with the components of the PDGF regulated signaling pathways such as ERKs or STATs in HTFs.[24] Stimulation of the control cells by PDGF resulted in enhanced phosphorylation of ERK1/2 (Fig 5A), STAT1 (Fig 5B), and STAT3 (Fig 5C). Preincubation of the cells with silibinin, diminished the PDGF-dependent phosphorylation of ERK1/2, STAT1, and STAT3 in a dose-dependent manner.

### Effect of silibinin on PDGF receptor

**Fig 6:**
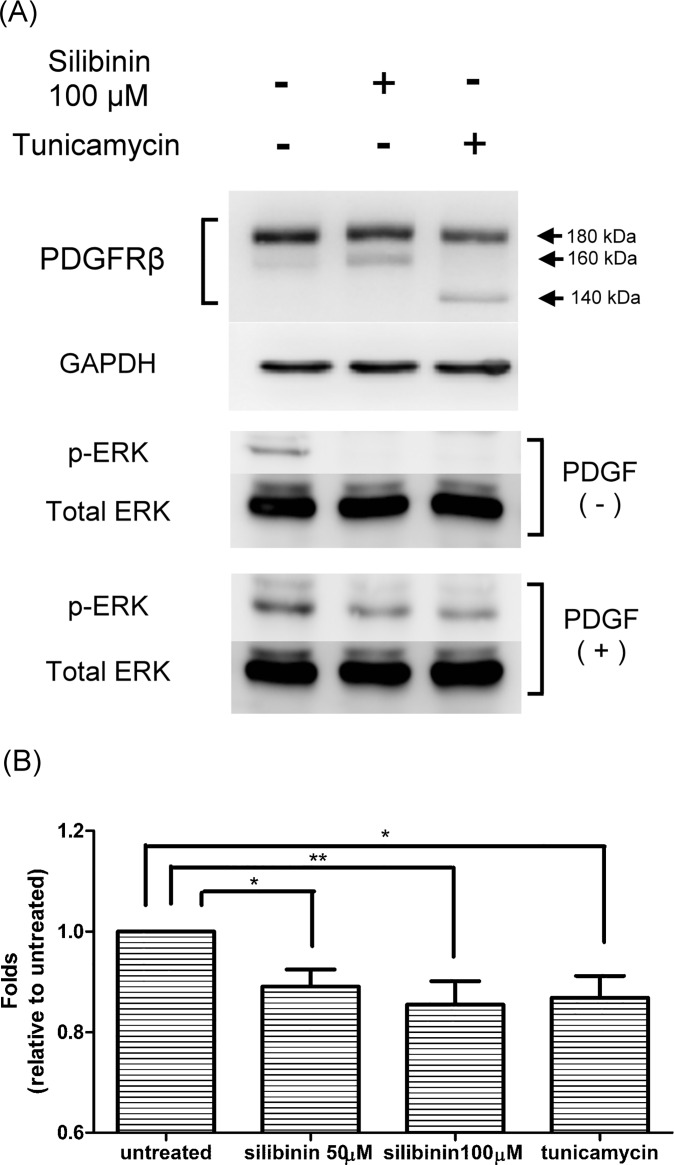
Effects of silibinin on platelet-derived growth factor receptor β (PDGFRβ) in human Tenon's fibroblasts (HTFs). (A) HTFs were pretreated with vehicle, 100 μM silibinin, or tunicamycin for 24 h. The cells were then treated with PDGF for 24 h. Whole cell lysates were prepared and analyzed via western blot by using antibodies directed against PDGFRβ, GAPDH, phosphorylated-ERK, and total ERK. (B) HTFs were pretreated with vehicle, 100 μM silibinin, or tunicamycin for 24 h. The cells were then treated with biotinylated rhPDGF-BB for 1 h. Flow cytometric analysis was used to analyze the ligand binding affinity of PDGF receptor on the surface of HTFs. Mean absorbance was measured and normalized to the control. The data are presented as means ± SEM of three independent experiments. Asterisks (* and **) indicate responses that are significantly different (p < 0.05 and p < 0.01, respectively).

We have shown herein that silibinin inhibited the proliferation of HTFs through two different signaling pathways, MAPK/ERK and STATs. On the basis of these findings, we hypothesized that the upstream PDGF receptor may be affected by silibinin. In our previous report, we showed that silibinin could inhibit the expression and activity of the cell surface receptor, ICAM-1, via downregulation of N-glycosylation [20]. It has also been reported that tunicamycin abolishes PDGF-mediated ERK1/2 signaling by blocking PDGFRβ maturation and its cell surface expression [25, 26]. We determined whether the N-linked glycosylation of PDGFRβ is affected by silibinin and whether it leads to the suppression of the downstream signaling pathways. As shown in Fig 6A, the mature form of PDGFRβ (molecular weight of 180 kDa) diminished in the HTFs treated with either 100 μM silibinin or tunicamycin. Conversely, the expression of the smaller immature isoforms of PDGFRβ with apparent molecular weights of 160 kDa and 140 kDa was observed in cells with silibinin and tunicamycin treatment, respectively. Upon stimulation by PDGF, the activation of ERK pathway was noted in cells with the mature isoform of PDGFRβ, which decreased significantly in those with the immature isoform of PDGFRβ. Preincubation of the cells with silibinin or tunicamycin diminished the PDGF-dependent phosphorylation of ERK. We further examined whether the ligand binding affinity of PDGF receptor on the surface of HTFs is affected by silibinin by using a fluorescence-based technology. As shown in Fig 6B, the mean fluorescence intensity, representing the ligand binding affinity of PDGF receptor, was significantly lower in HTFs treated with silibinin or tunicamycin than in control HTFs.

### MG132, a proteasome inhibitor, suppresses silibinin-induced hypo N-glycosylation of PDGFRβ

**Fig 7:**
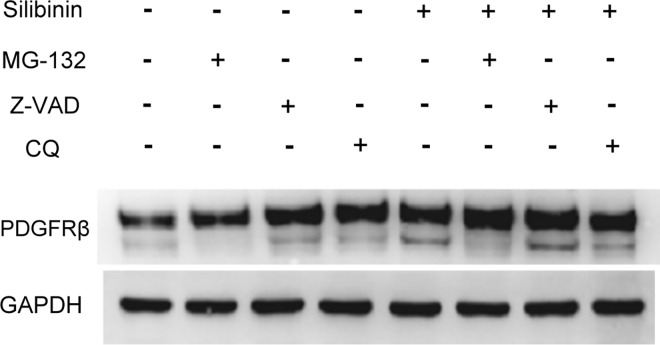
Effect of MG132 on silibinin-induced hypo N-glycosylated platelet-derived growth factor receptor β (PDGFRβ) in human Tenon's fibroblasts (HTFs). HTFs were pretreated with vehicle, MG132, Z-VAD, or CQ for 24 h. The cells were then treated with 100 μM silibinin for 24 h. Whole cell lysates were prepared and analyzed via western blot by using antibodies directed against PDGFRβ and GAPDH. Three independent experiments were performed.

Non-functional or defective proteins in cells are degraded by proteasome-, lysosome-, or caspase-mediated mechanisms. Using several pharmacological inhibitors such as the proteasome inhibitor (MG132), the lysosome inhibitor (CQ), or the pan-caspase inhibitor (Z-VAD), we next determined whether silibinin modification of PDGFRβ is associated with these mechanisms. As shown in Fig 7, the appearance of the hypo-glycosylated 160 kDa PDGFRβ increased in HTFs treated with 100 μM silibinin. However, HTFs treated with MG132, and not CQ or Z-VAD, failed to produce the hypo-glycosylated PDGFRβ with or without silibinin. The data obtained suggests the involvement of the proteasomal pathway in the production of the hypo-glycosylated PDGFRβ by silibinin.

## Discussion

In this study, we tested our hypothesis that silibinin inhibits PDGF-induced HTF proliferation in vitro and in vivo and to explore the underlying mechanisms. The results of the present study showed that treatment with silibinin at concentrations of 50 and 100 μM significantly suppressed the PDGF-induced proliferation of HTFs in a dose-dependent manner and delayed the cell cycle progression of HTFs through the G1-S transition without affecting cell viability. The antiproliferative effect of silibinin was also confirmed in a modified animal trabeculectomy model. We provided evidence that silibinin modulated the level of N-glycosylation of PDGFRβ and reduced the downstream signaling pathways, ERK and STATs. Finally, we showed that silibinin promoted the formation of hypo-glycosylated PDGFRβ in a proteasome-dependent manner.

In glaucoma filtering surgery, it is challenging to adequately regulate the healing process at the surgical site. Effective conjunctival wound healing is necessary to prevent bleb leakage [27]. Diminished fibrosis in both the subconjunctival space and the drainage pathway is also required to enhance the surgical success rate [28]. Until date, excessive scar formation has been a major reason for failure in glaucoma filtering surgery. Scar formation represents the termination of a normal wound healing process, which comprises several stages including hemostasis, inflammation, proliferation, and remodeling. In the proliferative phase, concurrently with angiogenesis, fibroblasts begin to accumulate and grow at the wound site [29]. Fibroblasts become the major cells in the wound, form the provisional extracellular matrix, and differentiate into myofibroblasts. At the same time, several growth factors or cytokines, such as PDGF, epidermal growth factor (EGF), or insulin-like growth factor, participate in the process to promote the proliferation of fibroblasts. Therefore, suppressing the proliferation of fibroblasts in the surgical site is one of the strategies for decreasing the bleb scarring in glaucoma filtering surgery. Mitomycin-C and 5-fluorouracil have been widely used for mitigating bleb fibrosis through their antiproliferative effects. However, complications have been reported in the use of these two agents [27, 30]. Silibinin has been ascribed anticancer potential because of its manifold inhibitory effects on tumor growth in various cancer cells. Using cultured cells and animal models, Shukla et al. showed that silibinin induces metabolic reprogramming, which diminishes the growth and cachectic properties of pancreatic cancer cells [31]. Momeny et al. showed the potential anti-cancer activity of silibinin to inhibit the proliferative and invasive characteristics of the epithelial ovarian cancer cells that show an autocrine heregulin/human epidermal growth factor receptor 3 (HRG/HER3) pathway [32]. In addition to inhibiting cancer cells, silibinin has been also reported to reduce PDGF-induced DNA synthesis and cell proliferation in hepatic stellate cells [33]. Our data also showed that silibinin effectively suppressed the proliferation of HTFs induced by PDGF without affecting cell viability. Furthermore, silibinin exerted inhibitory effects on the cells at the subconjunctival space in the modified animal model of trabeculectomy. These results suggest potential application of silibinin in trabeculectomy.

The cell cycle progression is a series of events that occur in cell replication. The cell cycle progression is tightly controlled by a subfamily of CDKs, the activity of which is regulated by several cyclins and CDK inhibitors.[34] Cyclin D is the first cyclin produced in the cell cycle, which is activated by extracellular signals such as PDGF. CDK4 binds to cyclin D and the active cyclin D-CDK4 complex is then formed. The complex phosphorylates the retinoblastoma susceptibility protein (Rb), which further dissociates from the E2F/DP1/Rb complex, an inhibitory element of the E2F responsive genes. As a result, E2F is activated, which results in transcription of various genes, such as cyclin E and cyclin A. Next, the cyclin E binds to CDK2, forming the cyclin E-CDK2 complex, which promotes the transition of cell from the G1 to S phase [35]. Silibinin has been reported to inhibit cell growth and induce G1 arrest in cell cycle progression of human prostate carcinoma, which was associated with decreased levels of cyclin D1, CDK4, and CDK6 and induction of Cip1/p21 and Kip1/p27, followed by increased binding with CDK2 [36, 37]. Similarly, exposure to silibinin resulted in a dose- and time-dependent growth inhibition, together with G1 arrest in bladder transitional cell carcinoma cells, which was also associated with upregulation of Cip1/p21 and Kip1/p27 and inhibition of CDKs and cyclins involved in G1 progression [38]. In this study, we observed that silibinin caused delayed cell cycle progression at the G1-S transition and concurrently suppressed the expression of cyclin D1 and CDK4 in PDGF-induced HTFs. These results again suggested that silibinin inhibited the PDGF regulated proliferation in HTFs.

PDGF-regulated cell proliferation is mediated by the PDGF-PDGFR signaling. PDGFR belongs to RTK receptor family [12]. PDGF binding to PDGFR activates its intrinsic tyrosine kinase activity, leading to receptor autophosphorylation and phosphorylation of tyrosine residues in various downstream signaling molecules, including ERK1/2, AKT, or STATs. Flavonoids, which bind to the ATP-binding site of several kinases, act as RTK inhibitors [39]. Silibinin is a flavonoid and impairs the EGF receptor regulated ERK signaling in various cancer cells [40]. Pre-incubation with silibinin was also reported to affect the early signaling events of the PDGFR activation including ERK and MEK phosphorylation [33]. Inhibitory effects of silibinin on STAT-1, STAT-3, and STAT-5 phosphorylation were also observed in orthotopic xenograft of PC-3 human prostate carcinoma [41]. In the present study, we show that the phosphorylation of molecules (ERK, STAT1, and STAT3), downstream of the PDGF-PDGFR signaling was reduced by silibinin in PDGF-stimulated HTFs. These results suggest that silibinin can effectively suppress the PDGF-PDGFR signaling.

Silibinin has been reported to regulate glucose metabolism and its beneficial effect in diabetics has been proposed [42–44]. N-linked glycosylation is an enzymatic process that attaches glycans to the asparagine residues of a protein, a modification, which is important for the structure and function of a protein. As we discussed previously, silibinin post-translationally alters the degree of N-linked glycosylation on the surface of ARPE-19 cells, an example of which is seen in the case of ICAM-1 protein [20]. PDGFR is one of the cell surface receptors and is a highly glycosylated protein in its mature form. Therefore, we explored the effect of silibinin on the degree of glycosylation of PDGFR. In the cells treated with silibinin, a hypo-glycosylated isoform of PDGFRβ increased, and the full-glycosylated isoform conversely decreased. Although non-glycosylated and immaturely glycosylated PDGFRβ can be activated by PDGF in vitro, only the fully glycosylated isoform has been found on the plasma membrane [45, 46]. In a previous study, tunicamycin or PDGF-BB overnight pretreatment was shown to induce the loss of ~180 kDa isoform of the receptor, in addition to loss of PDGF-BB signaling [26]. Our study showed that silibinin prevented the N-linked glycosylation and maturation of PDGFRβ as well as its activation by PDGF. We propose that the expression of PDGFRβ on the cell surface may diminish in response to silibinin, which further attenuates the PDGF-mediated responses. Indeed, binding of the PDGF on the cell surface was confirmed to be lower in cells after silibinin treatment.

The presence of defective proteins such as deglycosylated ones may be harmful to cells, and the cellular protein degradative systems, including the proteasome-, lysosome-, and/or caspase-mediated proteolytic pathways, may be activated to degrade such proteins [47]. Silibinin promotes the accumulation of the defective glycoproteins via disruption of N-glycosylation of proteins, including that of ICAM-1 and PDGFRβ, and may promote the activation of degradative pathways. In this study, we used the inhibitors of these pathways to evaluate this issue. Specifically, we found that the proteasome inhibitor, MG132, effectively inhibits silibinin-mediated expression of the hypoglycosylated PDGFRβ protein, but not the lysosome or caspase inhibitors. The MG132 is a natural triterpene proteasome inhibitor derived from a Chinese medicinal plant and inhibits 20S proteasome activity by covalently binding to the active site of the β subunits, which effectively blocks the proteolytic activity of the 26S proteasome complex [48]. It has been shown that the ubiquitin-proteasome pathway contributes to PDGFRβ protein turnover [49]. Our finding strongly indicated that the 26S proteasome pathway participates in promoting the production of the hypoglycosylated PDGFRβ protein in silibinin treated HTFs. Pretreatment with MG132 has been shown to attenuate the silibinin induced downregulation of FLICE-like inhibitory protein and survivin [50], which suggested that silibinin may reduce the protein levels via proteasome mediated degradation. Indole-3-carbinol combined with silibinin has also been shown to suppress cyclin D1 by enhancing its proteasomal degradation [51]. Therefore, the increased production of hypoglycosylated PDGFRβ by silibinin may be induced by enhancing the PDGFRβ turnover in a proteasome-dependent manner. It will be necessary in the future to perform ^35^S pulse-chase experiments to confirm this hypothesis.

## Conclusions

We showed that silibinin inhibits the PDGF-mediated cell proliferation and delays the cell cycle progression in vitro. We present mechanistic evidence that these effects of silibinin result from the downregulation of the PDGFR-regulated signaling, which may, at least partially, be caused by the increased expression of non-functional hypoglycosylated PDGFRβ. We also showed that the 26S proteasomal pathway was involved in this process. Silibinin also abrogated the cell proliferation at the subconjunctival space in the in vivo rat model of trabeculectomy. Our findings provide evidence that silibinin could be a therapeutic candidate as an adjunctive agent to decrease the failure rate of glaucoma filtering surgeries.
